# The Role of SETBP1 in Gastric Cancer: Friend or Foe

**DOI:** 10.3389/fonc.2022.908943

**Published:** 2022-07-11

**Authors:** Fujin Fang, Chengyou Liu, Qiong Li, Rui Xu, Tiantian Zhang, Xiaobing Shen

**Affiliations:** ^1^ Key Laboratory of Environmental Medical Engineering and Education Ministry, School of Public Health, Southeast University, Nanjing, China; ^2^ Department of Preventive Medicine, School of Public Health, Southeast University, Nanjing, China; ^3^ Department of Medical Engineering, Nanjing First Hospital, Nanjing, China; ^4^ Department of Clinical Laboratory, The Third People’s Hospital of Bengbu, Bengbu, China

**Keywords:** gastric cancer, SETBP1, TCGA, GSEA, prognostic marker

## Abstract

**Background:**

Gastric cancer (GC) remains a common disease with a poor prognosis worldwide. The SET binding protein 1 (SETBP1) has been implicated in the pathogenesis of several cancers and plays a dual role as an oncogene and a tumor suppressor gene. However, the role and underlying mechanism of SETBP1 in GC remain unclear.

**Materials and Methods:**

We used next-generation RNA sequencing (RNA-seq) data from The Cancer Genome Atlas (TCGA) to explore the correlation between SETBP1 expression and tumor progression. We then quantified SETBP1 expression in GC cells with real-time quantitative polymerase chain reactions (RT-qPCR). The chi-square test and logistic regression were used to assess the correlation between SETBP1 expression and clinicopathological features. Kaplan-Meier survival analysis and Cox proportional hazards regression model were used to assess the relationship between SETBP1 expression and survival. Finally, gene set enrichment analyses (GSEA) were used to examine GC-related signaling pathways in low and high SETBP1 expressing samples.

**Results:**

We found SETBP1 expression levels in GC tissues to be significantly lower than in adjacent non-tumor tissues in the TCGA database. In addition, SETBP1 expression differed significantly between groups classified by tumor differentiation. Furthermore, SETBP1 expression in diffuse-type GC was significantly higher than in intestinal-type GC. However, it did not differ significantly across pathological- or T-stage groups. RT-qPCR and comprehensive meta-analysis showed that SETBP1 expression is downregulated in GC cells and tissues. Interestingly, SETBP1 expression in poorly- or un-differentiated GC cells was higher than in well-differentiated GC cells. Moreover, the chi-square test and logistic regression analyses showed that SETBP1 expression correlates significantly with tumor differentiation. Kaplan–Meier curves indicated that patients with relatively high SETBP1 expression had a poor prognosis. Multivariate analyses indicated that SETBP1 expression might be an important predictor of poor overall survival in GC patients. GSEA indicated that 20 signaling pathways were significantly enriched in samples with high and low SETBP1 expression.

**Conclusion:**

SETBP1 may play a dual role in GC progression.

## Introduction

While the global incidence of gastric cancer (GC) is declining, it remains one of the most common causes of cancer-related death worldwide. There are three major subtypes of GC under the Lauren classification system but four subtypes under the World Health Organization (WHO) classification system (1, 2). Many GCs are associated with various pathogenic infections, including *Helicobacter pylori* and the Epstein-Barr virus (EBV) (3). While significant effort has been made in diagnosing and treating GC, the clinical outcomes of patients with advanced GC remain poor. The 5-year survival rate of GC patients is about 20−30% in most areas of the world (4). Only recently have researchers started to appreciate how heterogeneous GC is. Nevertheless, GC remains a deadly disease. Current treatment options or earlier detection strategies have not provided meaningful control of GC. Therefore, it remains crucial to provide novel strategies for reducing GC risk.

The SET binding protein 1 (SETBP1) gene encodes a large protein of 1542 amino acids localized in the cell nucleus and cytoplasm (5, 6). SETBP1 was originally found to interact with SET, a small protein inhibitor of tumor suppressors protein phosphatase 2 phosphatase activator (PP2A) and non-metastatic protein 23 H1 (NM23-H1) (7–9). It is now widely recognized as a tumor-associated gene (5), and alteration of SETBP1 expression has been implicated in several tissue-specific diseases (10–12), including cancer. The downregulation of SETBP1 expression has been reported to promote non-small cell lung cancer progression by inducing cellular epithelial-mesenchymal transition (EMT) and disordered immune status (5). In addition, SETBP1 accumulation has been found to induce P53 inhibition and genotoxic stress in neural progenitors underlying neurodegeneration in Schinzel-Giedion syndrome (13). Furthermore, overexpression of tripartite motif-containing 29 (*TRIM29*) is closely associated with adverse clinical outcomes in ovarian cancer and promotes the proliferation, migration, and invasion of ovarian cancer cells *via* the SETBP1/SET/PP2A carcinogenic signal axis (14).

SETBP1 mutations in patients have also received wide attention, especially in blood diseases (15–18). Carratt et al. demonstrated that mutant SETBP1 enhances activation of the mitogen-activated protein kinase 1 (MAPK) pathway by the proto-oncogene GTPase NRAS, promoting aggressive leukemia (19). In addition, SETBP1 mutations have been reported to drive leukemic transformation in ASXL transcriptional regulator 1 (ASXL1)-mutated myelodysplastic syndrome (MDS) (20). Moreover, a father and son diagnosed with atypical chronic myeloid leukemia (aCML) were both found to carry SETBP1 mutations, which are present in 24.3% of aCML patients (21). Therefore, SETBP1 mutations are believed to be a biomarker in disease diagnosis (22, 23). However, their relationship with GC progression remains unclear.

This study examines the relationship between SETBP1 expression and the clinicopathological features of GC patients to advance understanding of the underlying signaling pathways related to SETBP1 expression and GC progression. Our findings provide additional evidence of its suitability as a prognostic biomarker for GC.

## Materials and Methods

### Data Collection

The Cancer Genome Atlas (TCGA) is a landmark cancer genomics program that molecularly characterized over 20,000 primary cancers and matched normal samples representing 33 cancer types. The next-generation RNA sequencing (RNA-seq) data of 407 GC-related samples, 375 GC tissue and 32 adjacent non-tumor tissue samples, were retrieved from the TCGA database (https://portal.gdc.cancer.gov/). The details of the patients are provided in **Table S1**. We then compared the SETBP1 expression in cancer tissues and paracarcinoma tissues.

In addition, 11 datasets were downloaded from Gene Expression Omnibus (GEO) database: GSE13195, GSE13911, GSE26899, GSE27342, GSE29272, GSE33335, GSE37023, GSE54129, GSE63089, GSE64591, and GSE65801. These datasets include 761 GC tissue samples and 475 adjacent non-tumor tissue samples. Information on these GEO datasets is provided in **Table S2**.

These datasets were analyzed as previously described (24, 25) using the R software to assess the differential expression of SETBP1. The standardized mean difference (SMD) was calculated as the effect size for continuous outcomes. The accuracy of the results is reported as a 95% confidence interval (CI). The chi-squared (χ2) and heterogeneity (*I* ^2^) statistical tests were used to evaluate heterogeneity among datasets. The fixed effects were calculated using a fixed-effects model with *P*>0.05 or *I* ^2^<50% and a random-effects model otherwise.

### SETBP1 Expression Analysis and Survival Analysis

The Perl programming language was used to sort and merge the downloaded TCGA gene expression data. It was also used to obtain the survival data from clinical databases and eliminate the data without information on complete survival time and survival status. The *limma* package of the R statistical software was used to extract the mRNA expression data from the datasets. The *limma* and *beeswarm* packages were used to visualize the extracted data and plot scatter difference diagrams in R. SETBP1 expression data was matched with the complete survival information. A total of 319 patients with complete information that met our requirements were retained for analysis. Based on the median SETBP1 expression value, patients were divided into two groups: high SETBP1 expression and low SETBP1 expression. The *survival* package of the R software was used for visualization and to obtain the Kaplan-Meier survival curve.

A comprehensive meta-analysis was performed using Review Manager 5.3 software to assess differences in SETBP1 expression in the GEO database. The SMD with a 95% CI was used to calculate a combined value. The heterogeneity among the included datasets was evaluated by *χ*2 and *I* ^2^ statistical tests. A fixed-effects model was used to calculate the combined effect with *P*>0.05 or *I* ^2^<50%, and a random-effects model was used otherwise. The results are presented as a forest diagram.

### Cell Culture

Human gastric mucosal epithelial cells (GES-1) were maintained in Dulbecco’s Modified Eagle Medium (DMEM) (Gibco; Waltham, MA, USA) supplemented with 10% Fetal Bovine Serum (FBS) and 1% penicillin-streptomycin in a humidified incubator at 37°C with 5% CO_2_.

The human GC cell lines HGC-27, MGC-803, AGS, and MKN-28 were cultured in Roswell Park Memorial Institute (RPMI) 1640 (Gibco) or DMEM (Gibco), according to the manufacturer’s specifications and supplemented with 10% FBS and 1% penicillin-streptomycin at 37°C with 5% CO_2_.

### RT-PCR and RT-qPCR

RNA extraction was performed with TRIzol reagent (Invitrogen Life Technologies; Carlsbad, CA, USA). RNA samples were treated with gDNA remover reagent (GenStar) according to the manufacturer’s instructions to remove genomic DNA (gDNA). cDNA was synthesized using the RT-Phusion kit (Thermo Fisher Scientific; Waltham, MA, USA). Gene-specific mRNA levels were quantified using standard and quantitative RT-PCR (RT-qPCR) using the ΔΔCt method. The primer sequences are listed in **Table 1**.

**Table 1:** List of primers.

Gene	Sequence
SETBP1-F	5’-GCCAGCCGCAGTTGACAGTG-3’
SETBP1-R	5’-CCGCCGCTTGAACCTCTTCTTC-3’
GAPDH-F	5’-CAGGAGGCATTGCTGATGAT-3’
GAPDH-R	5’-GAAGGCTGGGGCTCATTT-3’

### Univariate and Multivariate Cox Regression Analyses

Univariate and multivariate analyses were performed using a Cox proportional hazard regression model. The hazard ratio (HR) and 95% CI were calculated, the independent predictive value of the clinicopathological features and SETBP1 expression on survival was quantitatively assessed, and the independent prognostic effect of SETBP1 on survival was estimated by adjusting for confounders. First, the Perl programming language was used to sort and merge the original clinical data; unknown or incomplete clinical information was removed. Then, the clinical data was matched with the SETBP1 expression data. Finally, the matched data were analyzed by univariate and multivariate Cox regression. According to the median SETBP1 expression value, the patients were divided into two groups: high SETBP1 expression and low SETBP1 expression. The data were analyzed and visualized using the *survival* and *survminer* packages of the R software with the *coxph* and *ggforest* commands.

### Gene Set Enrichment Analysis

The signaling pathways related to SETBP1 in GC were explored using GSEA (v.4.2.3). GSEA was performed between datasets with SETBP1 expression levels. The phenotype was determined by SETBP1 expression level based on the TCGA database. The annotated gene set was selected (c2.cp.kegg.v6.2.symbols.gmt) as the reference gene set. A total of 1000 gene sets were used in each analysis to identify pathways with significant differences. Each analysis was performed with 1000 permutations to identify pathways that differed significantly. The normalized enrichment score (NES), nominal *p*-value, and false discovery rate (FDR) *q*-value were used to assess the importance of the association between gene sets and pathways.

### Statistical Analysis

The differential expression of SETBP1 between GC tissues and adjacent non-tumor tissues was assessed with a Mann-Whitney U test. The differences in SETBP1 expression across multiple groups were compared using a Student’s *t*-test or Bonferroni correction. The χ2 test was used to evaluate associations between SETBP1 expression and clinicopathological features. Kaplan-Meier analysis and log-rank test were used to compare the significant differences in survival rates between the high- and the low-SETBP1 expression groups. A Cox proportional hazard regression model was used for univariate and multivariate survival analysis. Statistical analyses were performed with the IBM SPSS (v.23.0), GraphPad Prism (version 9.0), and R (version 2.15.3) software. Results with *P*<0.05 was considered statistically significant.

## Results

### The Differential Expression of SETBP1 in GC

**Figure 1:**
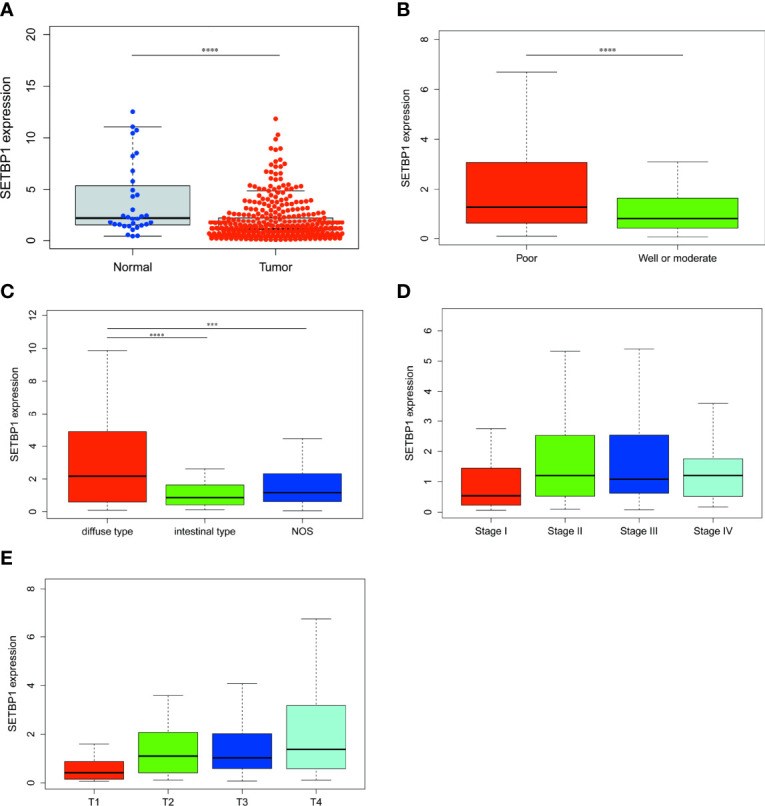
The differential expression of SETBP1 and its relationship with clinicopathological features based on TCGA data. **(A)** Differential expression of SETBP1 between GC tissues and adjacent non-tumor tissues. **(B)** The expression of SETBP1 is divided into groups by tumor differentiation **(C)**, Lauren classification **(D)**, pathological stage **(E)** and T stage. Poor, G3 (Poorly differentiated) and G4 (Undifferentiated); Well or moderate, G1 (Well differentiated) and G2 (Moderately differentiated). ****P < 0.0001, unpaired two-sided Student’s *t*-test. Error bars indicate mean and SD. There are no statistically significant differences across pathological stage or T stage groups. ****P < 0.0001, ***P < 0.001, Bonferroni correction was used for multiple comparison. NOS, Not otherwise specified.

RNA-seq data for 407 samples, representing 375 GC tissue and 32 adjacent non-tumor tissue samples, were downloaded from the TCGA database. We found SETBP1 expression to be significantly lower in GC tissues than in adjacent non-tumor tissues (*P*<0.0001; **Figure 1A**). In addition, SETBP1 expression was significantly different in groups classified according to tumor differentiation (*P*<0.0001), where SETBP1 expression was significantly higher in the poor group (G3, poorly differentiated; G4, undifferentiated) compared to the well or moderate groups (G1, well-differentiated; G2, moderately differentiated; **Figure 1B**). Moreover, SETBP1 expression in diffuse type GC was significantly higher than in intestinal type GC based on the Lauren classification system (*P*<0.0001; **Figure 1C**). However, SETBP1 expression did not differ statistically across pathological stages (**Figure 1D**) or T stages (**Figure 1E**).

### Confirmation of SETBP1 Differential Expression by RT-qPCR and SMD

**Figure 2:**
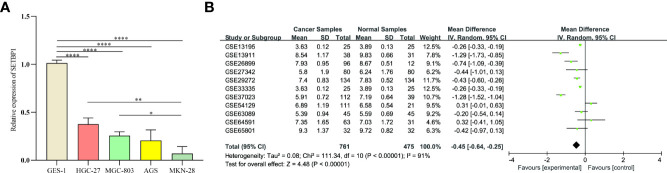
Real-time quantitative polymerase chain reaction analysis and forest plot of SETBP1 expression data from GEO datasets. **(A)** RT-qPCR analysis of SETBP1 mRNA expression in human gastric mucosal epithelial cells (GES-1) and human GC cell lines (HGC-27, MGC-803, AGS and MKN-28). Data are the mean ± s.d. of three independent experiments. ****P < 0.0001, **P < 0.01, *P < 0.05, unpaired two-sided Student’s *t*-test. **(B)** The pooled of SETBP1 is -0.45 (95% CI, -0.64–0.25) by the random effects model. SMD, standard mean difference; CI, confidence interval.

>To confirm SETBP1 differential expression in the TCGA database, RT-qPCR was used to evaluate SETBP1 expression in five cell lines. SETBP1 expression in GC cells (HGC-27, MGC-803, AGS, and MKN-28) was significantly lower than that in GES-1 cells (*P*<0.0001; **Figure 2A**). Interestingly, SETBP1 expression in well-differentiated GC cells (MKN-28) was significantly lower than in poorly differentiated (MGC-803; *P*<0.05) and undifferentiated (HGC-27; *P*<0.01) GC cells (**Figure 2A**). Moreover, the confirmation of SETBP1 expression was performed using the GEO datasets, providing an *I^2^* value of 91% (*P*<0.00001) and a combined SMD for SETBP1 of -0.45 based on the random-effects model (95% CI: -0.64–0.25; **Figure 2B**), indicating that SETBP1 is expressed at low levels in GC.

### Highly Expressed Genes Are Associated With Poor Overall Survival in GC

**Figure 3:**
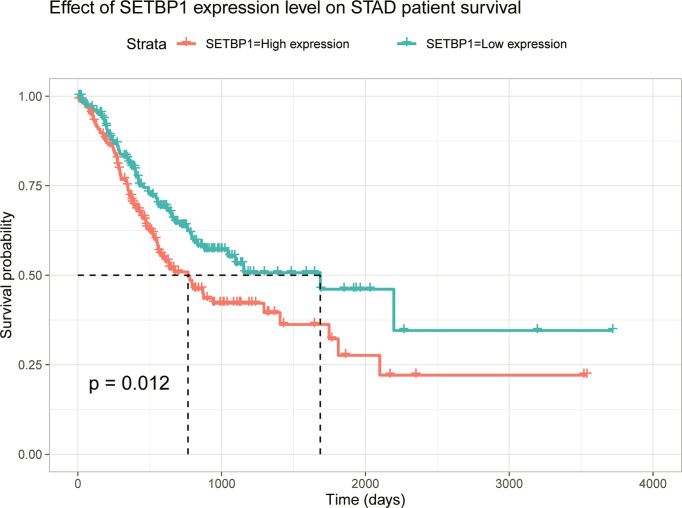
Kaplan-Meier curve for the relationship between SETBP1 expression and the prognosis of GC patients based on the TCGA database (*P*=0.012).

The prognosis of high SETBP1 expression in GC was assessed based on the TCGA using Kaplan-Meier risk estimation. High SETBP1 expression was more significantly correlated with poor overall survival than low SETBP1 expression (*P*=0.012; **Figure 3**). The 50% survival probability in the high SETBP1 expression group was about half the number of survival days in the low SETBP1 expression group (**Figure 3**).

### The Relationship Between SETBP1 Expression and Clinicopathological Features

We assessed the relationship between SETBP1 expression and clinicopathological features, finding that high SETBP1 expression was significantly correlated with tumor differentiation (*P*=0.001; **Table 2**). Logistic regression analyses indicated that higher SETBP1 expression in GC was significantly associated with age (odds ratio [OR]=1.541 for ≥65 vs. <65, *P*=0.041), tumor differentiation (OR = 0.490 for poor vs. well or moderate, *P*=0.001), pathological stage (OR=2.679 for stage II vs. stage I, *P*=0.005; OR=2.118 for stage III vs. stage I, *P*=0.026; OR=2.912 for stage IV vs. stage I, *P*=0.015), T stage (OR=3.750 for T2 vs. T1, *P*=0.029; OR=3.329 for T3 vs. T1, *P*=0.039; OR=5.625 for T4 vs. T1, *P*=0.004; **Table 3**).

**Table 2:** The relationship between SETBP1 expression and clinicopathological features in GC.

Clinicopathological features	SETBP1 expression		Total (*N*)	*P*-value
	High (*n*=160)	Low (*n*=159)		
**Age**				
<65 years	74 (55%)	60 (45%)	134	0.123
≥65 years	86 (47%)	99 (53%)	185	
**Gender**				
Male	95 (48%)	104 (52%)	199	0.266
Female	65 (54%)	55 (46%)	120	
**Tumor differentiation**				
Well or moderate	45 (39%)	72 (61%)	117	**0.001**
Poor	115 (57%)	87 (43%)	202	
**Pathological stage**				
I–II	68 (47%)	77 (53%)	145	0.288
III–IV	92 (53%)	82 (47%)	174	
**T classification**				
T1–T2	34 (43%)	46 (57%)	80	0.114
T3–T4	126 (53%)	113 (47%)	239	
**Lymph node metastasis**				
Negative	47 (47%)	54 (53%)	101	0.378
Positive	113 (52%)	105 (48%)	218	
**Distant metastasis**				
No	148 (68%)	149 (32%)	217	0.670
Yes	12 (55%)	10 (45%)	22	

The bold values mean statistically significant.

**Table 3:** SETBP1 expression correlated with clinicopathological features in GC.

Clinicopathological features	Total (*N*)	Odds ratio in SETBP1 expression	*P*-value
**Age**			
≥65 vs. <65	371	1.541 (1.018-2.338)	**0.041**
**Gender**			
Male vs. female	375	0.863 (0.565-1.316)	0.493
**Tumor differentiation**			
Poor vs. Well or moderate	366	0.490 (0.319-0.748)	**0.001**
**Pathological stage**			
Stage II vs. stage I	164	2.679 (1.363-5.426)	**0.005**
Stage III vs. stage I	203	2.118 (1.108-4.172)	**0.026**
Stage IV vs. stage I	91	2.912 (1.241-7.042)	**0.015**
**T classification**			
T2 vs. T1	99	3.750 (1.236-14.046)	**0.029**
T3 vs. T1	187	3.329 (1.153-12.050)	**0.039**
T4 vs. T1	119	5.625 (1.885-20.851)	**0.004**
**Lymph node metastasis**			
Positive vs. negative	357	1.256 (0.802-1.972)	0.321
**Distant metastasis**			
Yes vs. no	355	1.304 (0.577-3.020)	0.525

The bold values mean statistically significant.

### Univariate and Multivariate Analysis of the Relationship Between SETBP1 Expression and GC

**Figure 4:**
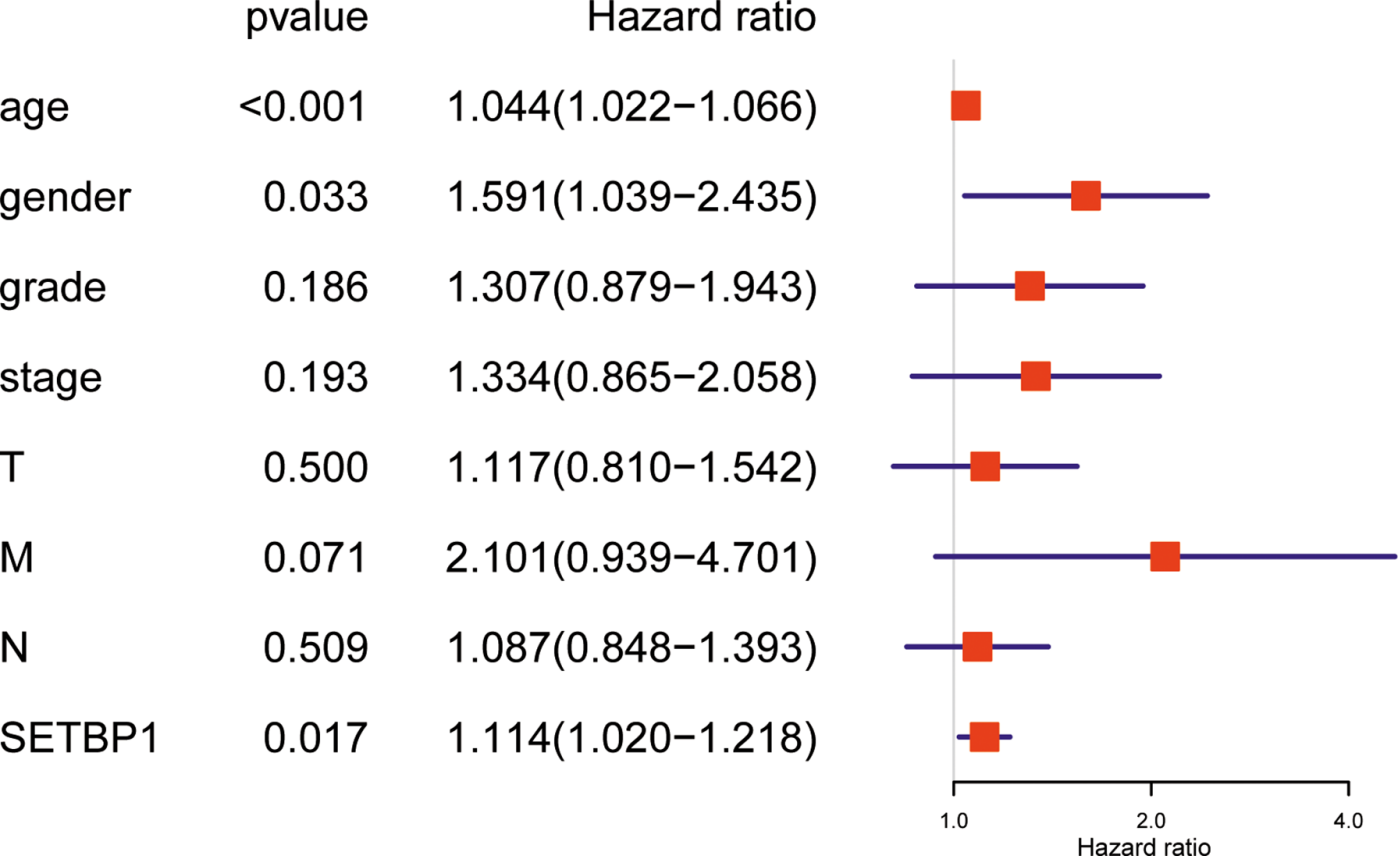
Forest plot for the multivariate Cox proportional hazard regression model. SETBP1 could act as an independent predictor of poor survival rate (HR, 1,114; 95% CI, 1.020-1.218; *P* = 0.017) in different grading of GC patients. HR, hazard ratio; CI, confidence interval.

Overall survival was significantly correlated with SETBP1 expression in GC patients. Therefore, univariate and multivariate analyses of the relationship between SETBP1 expression in GC patients were performed to jointly assess the effects of SETBP1 expression and clinicopathological features on the overall survival. The univariate analysis showed that age (HR=1.027; 95% CI: 1.008-1.047; *P*=0.006), pathological stage (HR=1.535; 95% CI: 1.221-1.931; *P*=0.000), T stage (HR=1.298; 95% CI: 1.023-1.645; *P*=0.032), M stage (HR=2.048; 95% CI: 1.096-3.827; *P*=0.025), N stage (HR=1.267; 95% CI: 1.069-1.502; *P*=0.006) were important survival predictors (**Table 4**). Furthermore, multivariate analysis showed that increased SETBP1 expression might be a prominent freestanding predictor of poor overall survival in GC (HR=1.114; 95% CI: 1.020-1.218; *P*=0.017) (**Table 4**; **Figure 4**).

**Table 4:** Univariate and multivariate analysis of the relationship between SETBP1 expression and GC patients.

Clinicopathological features	Univariate analysis			Multivariate analysis		
	HR	95% CI	*P*-value	HR	95% CI	*P*-value
**Age**	1.027	1.008-1.047	**0.006**	1.044	1.022-1.066	**0.000**
**Gender**	1.484	0.980-2.247	0.062	1.591	1.039-2.435	**0.033**
**Grade**	1.368	0.947-1.977	0.095	1.307	0.879-1.943	0.186
**Pathological stage**	1.535	1.221-1.931	**0.000**	1.334	0.865-2.058	0.193
**T**	1.298	1.023-1.645	**0.032**	1.117	0.810-1.542	0.500
**M**	2.048	1.096-3.827	**0.025**	2.101	0.939-4.701	0.071
**N**	1.267	1.069-1.502	**0.006**	1.087	0.848-1.393	0.509
**SETBP1**	1.054	0.976-1.139	0.181	1.114	1.020-1.218	**0.017**

HR, hazard ratio; CI, confidence interval. The bold values mean statistically significant.

### Identify the Signaling Pathways Associated With SETBP1

**Figure 5:**
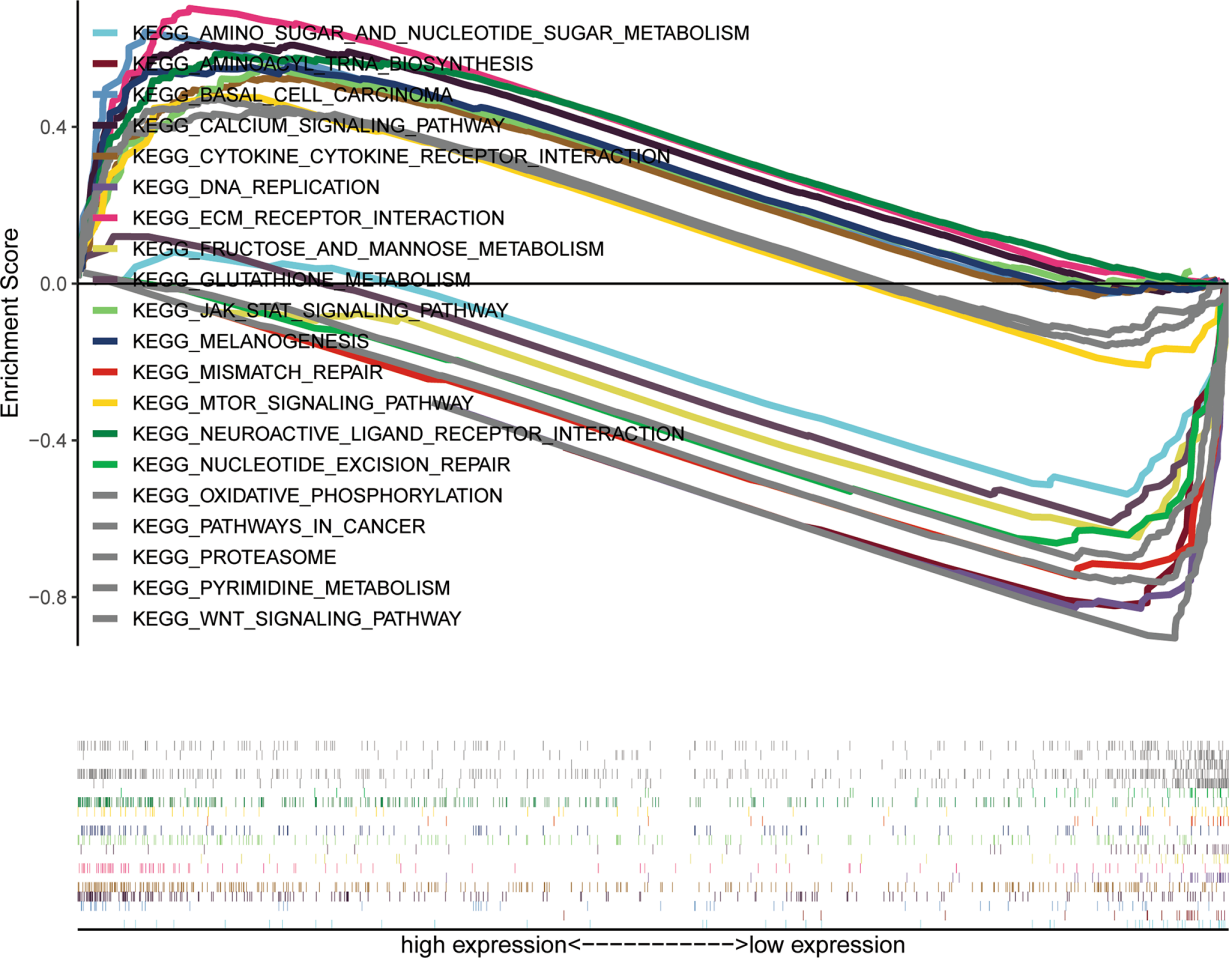
A combined enrichment maps from genomic enrichment analysis, enrichment scores and genomes were included.

This study explored the function of SETBP1 and its associated signaling pathways through GSEA in the TCGA database based on NES, FDR *q*-values, and nominal *p*-values to identify significantly enriched signaling pathways. We identified 10 and 10 signaling pathways significantly enriched for genes differentially expressed in the high and low SETBP1 expression groups, respectively. The neuroactive ligand receptor interaction, calcium signaling pathway, jak stat signaling pathway, extracellular matrix (ECM) receptor interaction, pathways in cancer, Wnt signaling pathway, mammalian target of rapamycin (mTOR) signaling pathway, basal cell carcinoma, melanogenesis, and cytokine-cytokine receptor interaction gene sets were significantly enriched in the high SETBP1 expression group. In addition, the pyrimidine metabolism, oxidative phosphorylation, proteasome, glutathione metabolism, aminoacyl tRNA biosynthesis, fructose and mannose metabolism, DNA replication, nucleotide excision repair, mismatch repair, amino sugar and nucleotide sugar metabolism gene sets were significantly enriched in the low SETBP1 expression group (**Table 5**; **Figures 5**, **S1**, and **S2**).

**Table 5:** Gene sets enriched in the SETBP1 expression phenotype.

SETBP1 expression level	Gene set name	NES	NOM *p*-value	FDR *q*-value
High-SETBP1 expression	KEGG_NEUROACTIVE_LIGAND_RECEPTOR_INTERACTION	2.34	0.000	0.001
	KEGG_CALCIUM_SIGNALING_PATHWAY	2.28	0.000	0.001
	KEGG_JAK_STAT_SIGNALING_PATHWAY	2.06	0.000	0.011
	KEGG_ECM_RECEPTOR_INTERACTION	2.05	0.004	0.009
	KEGG_PATHWAYS_IN_CANCER	1.77	0.011	0.433
	KEGG_WNT_SIGNALING_PATHWAY	1.62	0.019	0.083
	KEGG_MTOR_SIGNALING_PATHWAY	1.57	0.045	0.096
	KEGG_BASAL_CELL_CARCINOMA	2.05	0.000	0.008
	KEGG_MELANOGENESIS	2.02	0.000	0.010
	KEGG_CYTOKINE_CYTOKINE_RECEPTOR_INTERACTION	1.91	0.006	0.020
Low-SETBP1 expression	KEGG_PYRIMIDINE_METABOLISM	-2.28	0.000	0.001
	KEGG_OXIDATIVE_PHOSPHORYLATION	-2.22	0.000	0.001
	KEGG_PROTEASOME	-2.238	0.000	0.000
	KEGG_GLUTATHIONE_METABOLISM	-2.06	0.002	0.004
	KEGG_AMINOACYL_TRNA_BIOSYNTHESIS	-2.03	0.000	0.006
	KEGG_FRUCTOSE_AND_MANNOSE_METABOLISM	-1.97	0.002	0.011
	KEGG_DNA_REPLICATION	-2.00	0.000	0.008
	KEGG_NUCLEOTIDE_EXCISION_REPAIR	-1.91	0.012	0.016
	KEGG_MISMATCH_REPAIR	-1.87	0.008	0.017
	KEGG_AMINO_SUGAR_AND_NUCLEOTIDE_SUGAR_METABOLISM	-1.66	0.035	0.056

NES, normalized enrichment score; NOM, nominal; FDR, false discovery rate.

## Discussion

SETBP1 expression has been extensively studied in various diseases, including cancers, where it is abnormal compared with normal tissues. These studies have involved several cancer types, including non-small cell lung cancer (5), hematologic malignancies (26), colorectal cancer (27), ovarian cancer (14), breast cancer (12), and gastric cancer (28, 29). However, SETBP1 appears to play a dual role in different cancer types. It has been shown that SETBP1 is highly expressed in some cancers, driving carcinogenesis. SETBP1 has been reported to be overexpressed in acute myeloid leukemia (AML), protecting the SET protein from proteasome degradation, and leading to increased levels of full-length SET protein. Patients with SETBP1 overexpression have a significantly shorter overall survival (30). In addition, TRIM29 can facilitate SETBP1 transcriptional activation *via* the vascular endothelial zinc finger 1 (VEZF1) transcription factor, promoting ovarian cancer progression (14). Moreover, SETBP1 was identified as an oncogene contributing to breast cancer development (12).

Conversely, lower SETBP1 expression was found to promote non-small cell lung cancer progression by inducing cellular EMT and disordered immune status (5). It has also been reported that SETBP1 expression is similar or lower in colorectal cancer tissue compared to normal colonic mucosa (27). Furthermore, SETBP1 mutations have been found to be involved in serval cancers, including myeloid neoplasms (15), lung cancer (31, 32) and colorectal cancer (27). Therefore, the role of SETBP1 in cancer remains controversial and in GC remains obscure.

Our study uncovered a relationship between SETBP1 and GC. We showed that SETBP1 expression was significantly lower in GC tissues than in adjacent non-tumor tissues in the TCGA database (**Figure 1A**). However, the small number of normal tissue samples (32) may increase the risk of bias due to sampling error. We performed a meta-analysis to confirm low SETBP1 expression in GC based on the GEO database (**Figure 2B**), finding the TCGA and GEO results to be consistent. Furthermore, we showed that SETBP1 expression in GC cell lines (HGC-27, MGC-803, AGS, and MKN-28) was significantly lower than that in the GES-1 cell line (**Figure 2A**), consistent with earlier studies (5, 27). Therefore, the findings of this study indicate that SETBP1 may act as a tumor suppressor gene.

Interestingly, the Kaplan-Meier survival analysis showed that the high SETBP1 expression group had a worse overall GC survival prognosis than the low SETBP1 expression group (**Figure 3**). In addition, the multivariate analysis showed that high SETBP1 expression was related to poor overall survival and other clinicopathological features (**Figure 4**; **Table 4**). Interestingly, these results were consistent with those of our previous work (**Figure 1B**), with a worse prognosis associated with a lower grade (33). These findings were confirmed in our human GC cell experiments. SETBP1 expression in poorly differentiated (MGC-803) or undifferentiated (HGC-27) GC cells was higher than in well-differentiated GC cells (MKN-28). In addition, intestinal type cancers are common and reported to have a better prognosis than diffuse type cancers. In this study, SETBP1 expression in diffuse-type GC was significantly higher than in intestinal-type GC. Therefore, lower SETBP1 expression may confer better overall survival in GC patients, indicating that SETBP1 may act as an oncogene. Consequently, our findings appear contradictory and indicate that SETBP1 may have a dual role in GC development.

The SETBP1-associated signaling pathways in GC were identified *via* GSEA, with 10 and 10 KEGG pathways enriched in high- and low-SETBP1 expression groups, respectively (**Table 5**). In the high SETBP1 expression group, the neuroactive ligand receptor interaction signaling pathway is related to tumor TNM stage, lymph node metastasis, and poor prognosis with GC (34). In addition, it can trigger the downstream phosphoinositide 3-kinase (PI3K)/protein kinase B (Akt) signal pathway that is often associated with cancer (35). The calcium signaling pathway plays a role in multiple cellular processes involved in metabolism, secretion, fertilization, proliferation, and smooth muscle contraction (36–38). The Janus kinase (JAK)/signal transducer and activator of transcription (STAT) signaling pathway is universally expressed and involved in many important biological processes, including cell proliferation, differentiation, apoptosis, and immune regulation (39). The ECM-receptor interaction pathway plays an important role in the tumor shedding, adhesion, degradation, movement, and hyperplasia processes (40). Similar to the PI3K/Akt signal pathway, the pathways in cancer, basal cell carcinoma signaling pathway (41), mTOR signaling pathway (42), Wnt signaling pathway (43) and melanogenesis signaling pathway (44) are common cancer-related pathways. The cytokine receptor signaling pathway is an immune-related signaling pathway that affects tumor progression (45). Therefore, SETBP1 may promote tumor progression by regulating various signaling pathways.

In the low SETBP1 expression group, the pyrimidine metabolism signaling pathway is critical for the generation of pyrimidines for DNA replication, RNA synthesis, and cellular bioenergetics. Increased nucleotide metabolism supports the uncontrolled growth of tumors (46). Oxidative phosphorylation is upregulated in some cancers, including leukemia, lymphoma, and pancreatic ductal adenocarcinoma (47). Proteasome (48), glutathione metabolism (49), aminoacyl tRNA biosynthesis (50), fructose and mannose metabolism (51), and amino sugar and nucleotide sugar metabolism (52, 53) are all common metabolism-related signaling pathways. DNA replication (54), nucleotide excision repair (55), and mismatch repair (56) are nucleic acid biosynthesis-related signaling pathways contributing to the cell cycle. Therefore, SETBP1 can function either as an oncoprotein or tumor suppressor, and further studies on its role in GC are required.

GC is a heterogeneous disease that affects a large number of individuals per year and remains an unmet clinical problem (57). Due to the high mortality of GC, various studies have sought to identify biomarkers to support earlier detection and improve the survival status of GC patients. In this study, we have shown that SETBP1 may have a dual role in GC development. However, our study had some limitations. Further studies with larger sample sizes and on the possible dynamic regulatory mechanism by which SETBP1 contributes to GC are required to determine whether SETBP1 functions as an oncoprotein or tumor suppressor in GC. Additional studies are needed to understand how to treat GC and cancer in general. Hopefully, as research progresses, their findings will provide important insights into these critical issues.

## Data Availability Statement

Publicly available datasets were analyzed in this study. This data can be found here: TCGA database (https://portal.gdc.cancer.gov/). GEO: GSE13195, GSE13911, GSE26899, GSE27342, GSE29272, GSE33335, GSE37023, GSE54129, GSE63089, GSE64591, GSE65801.

## Author Contributions

FF contributed to the conception and design of the current study and was responsible for drafting the manuscript. In addition, FF also completed the experimental part. CL, QL, RX, and TZ contributed to process the data and revised the manuscript. The corresponding author (XS) is responsible for ensuring that the descriptions are accurate and agreed by all authors. All authors contributed to the article and approved the submitted version.

## Conflict of Interest

The authors declare that the research was conducted in the absence of any commercial or financial relationships that could be construed as a potential conflict of interest.

## Publisher’s Note

All claims expressed in this article are solely those of the authors and do not necessarily represent those of their affiliated organizations, or those of the publisher, the editors and the reviewers. Any product that may be evaluated in this article, or claim that may be made by its manufacturer, is not guaranteed or endorsed by the publisher.

## Supplementary Material

Supplementary Figure 1 The significantly enriched signaling pathways associated with the increased SETBP1 expression. **(A)** Neuroactive ligand receptor interaction, **(B)** calcium signaling pathway, **(C)** jak stat signaling pathway, **(D)** ECM receptor interaction, **(E)** pathways in cancer, **(F)** Wnt signaling pathway, **(G)** mTOR signaling pathway, **(H)** basal cell carcinoma, **(I)** melanogenesis, **(J)** cytokine cytokine receptor interaction.

Supplementary Figure 2 The significantly enriched signaling pathways associated with the decreased SETBP1 expression. **(A)** pyrimidine metabolism, **(B)** oxidative phosphorylation, **(C)** proteasome, **(D)** glutathione metabolism, **(E)** aminoacyl tRNA biosynthesis, **(F)** fructose and mannose metabolism, **(G)** DNA replication, **(H)** nucleotide excision repair, **(I)** mismatch repair, **(J)** amino sugar and nucleotide sugar metabolism.

Supplementary Table 1 Clinicopathological features of patients with GC. Tumor, node, metastasis (TNM) classification.

Supplementary Table 2 Relevant information of the selected GEO series dataset.
