# Supplementary figures and images for: The Role of SETBP1 in Gastric Cancer: Friend or Foe
Source: Front Oncol. 2022 Jul 11;12:908943. doi: 10.3389/fonc.2022.908943 (PMC9309353; doi:10.3389/fonc.2022.908943)

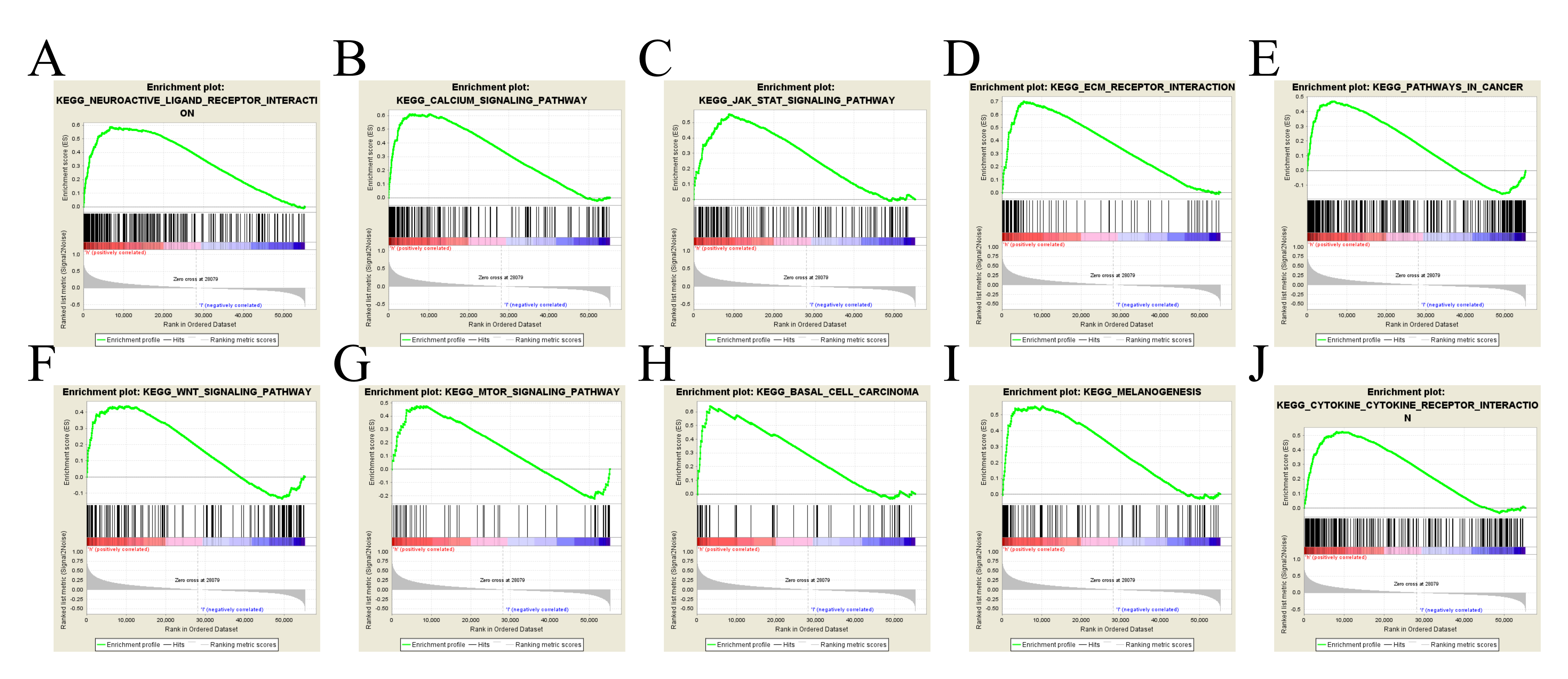

Supplement: Supplementary Figure 1 — The significantly enriched signaling pathways associated with the increased SETBP1 expression. (A) Neuroactive ligand receptor interaction, (B) calcium signaling pathway, (C) jak stat signaling pathway, (D) ECM receptor interaction, (E) pathways in cancer, (F) Wnt signaling pathway, (G) mTOR signaling pathway, (H) basal cell carcinoma, (I) melanogenesis, (J) cytokine cytokine receptor interaction. [file Image_1.tif]

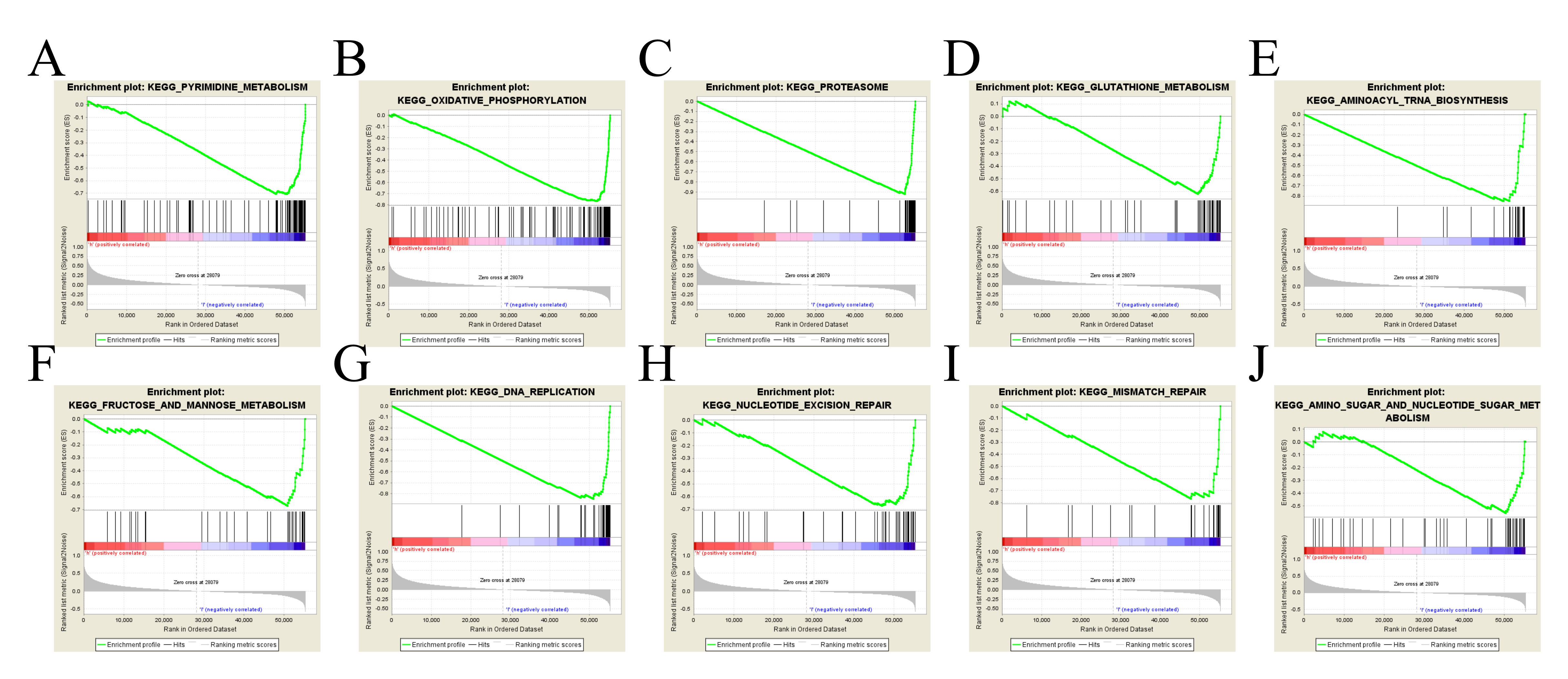

Supplement: Supplementary Figure 2 — The significantly enriched signaling pathways associated with the decreased SETBP1 expression. (A) pyrimidine metabolism, (B) oxidative phosphorylation, (C) proteasome, (D) glutathione metabolism, (E) aminoacyl tRNA biosynthesis, (F) fructose and mannose metabolism, (G) DNA replication, (H) nucleotide excision repair, (I) mismatch repair, (J) amino sugar and nucleotide sugar metabolism. [file Image_2.tif]
