# Supplementary material for: The Role of SETBP1 in Gastric Cancer: Friend or Foe
Source: Front Oncol. 2022 Jul 11;12:908943. doi: 10.3389/fonc.2022.908943 (PMC9309353; doi:10.3389/fonc.2022.908943)
Supplement: Supplementary Table 1 — Clinicopathological features of patients with GC. Tumor, node, metastasis (TNM) classification. [file Table_1.pdf]

Table S1. Clinicopathological features of patients with GC.

**Clinicopathological features**	**classification**	**Patients (n=375)**	**Percentages (%)**
Age	<65 years	155	41.33
	≥65 years	216	57.60
	Unknown	4	1.07
Gender	Male	241	64.27
	Female	134	35.73
Grade	G1	10	2.67
	G2	137	36.53
	G3	219	58.40
	GX	9	2.40
Pathological stage	I	53	14.13
	II	111	29.60
	III	150	40.00
	IV	38	10.13
	Unknown	23	6.14
T	T1	19	5.07
	T2	80	21.33
	T3	168	44.80
	T4	100	26.67
	TX	8	2.13
N	N0	111	29.60
	N1	97	25.87
	N2	75	20.00
	N3	74	19.73
	NX	16	4.27
	Unknown	2	0.53
M	M0	330	88.00
	M1	25	6.67
	MX	20	5.33
Vital status	Alive	244	65.07
	Death	131	34.93

Tumor, node, metastasis (TNM) classification.
