# Supplementary material for: The Role of SETBP1 in Gastric Cancer: Friend or Foe
Source: Front Oncol. 2022 Jul 11;12:908943. doi: 10.3389/fonc.2022.908943 (PMC9309353; doi:10.3389/fonc.2022.908943)
Supplement: Supplementary Table 2 — Relevant information of the selected GEO series dataset. [file Table_2.pdf]

Table S2. Relevant information of the selected GEO series dataset

GEO datasets	Platform	Sample	Total (*N*)	Country	Year
GSE13195	GPL5175	GC	25	China	2009
		Non-GC	25		
GSE13911	GPL570	GC	38	Italy	2008
		Non-GC	31		
GSE26899	GPL6947	GC	96	USA	2016
		Non-GC	12		
GSE27342	GPL5175	GC	80	USA	2011
		Non-GC	80		
GSE29272	GPL96	GC	134	USA	2013
		Non-GC	134		
GSE33335	GPL5175	GC	25	China	2012
		Non-GC	25		
GSE37023	GPL96	GC	112	Singapore	2012
		Non-GC	39		
GSE54129	GPL570	GC	111	China	2017
		Non-GC	21		
GSE63089	GPL5175	GC	45	China	2014
		Non-GC	45		
GSE64591	GPL570	GC	63	USA	2015
		Non-GC	31		
GSE65801	GPL14550	GC	32	China	2015
		Non-GC	32		
